# PDPN contributes to constructing immunosuppressive microenvironment in IDH wildtype glioma

**DOI:** 10.1038/s41417-022-00550-6

**Published:** 2022-11-25

**Authors:** Xuya Wang, Xisen Wang, Jiabo Li, Jianshen Liang, Xiao Ren, Debo Yun, Jie Liu, Jikang Fan, Yiming Zhang, Jinhao Zhang, Xiude Ren, Hao Zhang, Guanjie Shang, Jinzhang Sun, Lulu Chen, Lei Chen, Tao Li, Luqing Tong, Chen Zhang, Shengping Yu, Xuejun Yang

**Affiliations:** 1 Department of Neurosurgery, Tianjin Medical University General Hospital, Tianjin, China; 2 Laboratory of Neuro-oncology, Tianjin Neurological Institute, Tianjin, China; 3 Second Clinical Medical College, North Sichuan Medical College, Nanchong, China; 4 Department of Neurosurgery, Nanchong Central Hospital, Nanchong, China; 5 Department of Neurosurgery, The First Affiliated Hospital, Zhejiang University School of Medicine, Hangzhou, Zhejiang China; 6 Department of Neurosurgery, Beijing Tsinghua Changgung Hospital, Beijing, China

**Keywords:** CNS cancer, Tumour immunology, Tumour biomarkers

## Abstract

The tumor immunosuppressive microenvironment (IME) significantly affects tumor occurrence, progression, and prognosis, but the underlying molecular mechanisms remain to make known. We investigated the prognostic significance of PDPN and its role in IME in glioma. Weighted gene co-expression network analysis (WGCNA) found PDPN closely related to IDH wildtype status and higher immune score. Correlation analysis suggested PDPN was highly positively relevant to immune checkpoints expression and immune checkpoints block responding status. Correlation analysis together with verification in vitro suggested PDPN highly positively relevant tumor-associated neutrophils (TANs) and tumor-associated macrophages (TAMs). Least absolute shrinkage and selection operator (LASSO) regression employed to develop the prediction model with TANs and TAMs markers showed that high risk scores predicted worse prognosis. We highlight that PDPN overexpression is an independent prognostic indicator, and promotes macrophage M2 polarization and neutrophil degranulation, ultimately devotes to the formation of an immunosuppressive tumor microenvironment. Our findings contribute to re-recognizing the role of PDPN in IDH wildtype gliomas and implicate promising target therapy combined with immunotherapy for this highly malignant tumor.

## Introduction

Glioma refers to a notorious malignant solid tumor with the characteristic of the extremely immunosuppressive microenvironment. Many traditional therapies and the emerging immune therapies that show promising effects on other solid tumors have failed to slow down the progress of glioma [1, 2]. IDH mutation is an important diagnostic marker for adult diffuse glioma. Gliomas harboring mutations in IDH have the CpG island methylator phenotype and significantly longer patient survival time than IDH wildtype tumors [3]. Interestingly, IDH-mutant gliomas are infiltrated by less PD-1 expressing T cells and less immunosuppressive M2 Macrophages than those found in IDH wildtype gliomas [4, 5]. According to the latest WHO classification of CNS tumors, IDH wildtype diffuse glioma with histological appearance of necrosis or microvascular proliferation, or genetic alterations as TERT promoter mutation, EGFR amplification, or Chr 7 Gain and Chr 10 Loss should be diagnosed as “Glioblastoma, IDH wildtype” [6–8]. This type of glioma has common core signal transduction pathways, including RB, P53, TERT, and RTK/RAS/PI3K etc. with a high degree of heterogeneity [9]. The tumor microenvironment and non-tumor cells can also strongly influence the gene expression and transcriptional profiles of glioblastoma, in return, which promotes chemotaxis and activation of various cell populations to form the microenvironments [10]. Further studies on the interplay between glioma cells with the IME, especially in “Glioblastoma, IDH wildtype”, are urgent to be carried out to identify more molecular biomarkers, which are promising to provide guidance for targeted therapy and immunotherapy.

PDPN is a cell surface protein found expressed in different tissues throughout the body [11, 12]. During the embryonic development, PDPN in the neuroepithelium interacts with CLEC-2 on the platelets, mediating platelet adhesion, aggregation, and secretion to guide the maturation and integrity of the developing vasculature [13]. Intriguingly, PDPN has been implicated in malignant progression and invasion of a variety of human cancers, including gliomas [14–17]. Knockdown of PDPN in glioma cells resulted in decreased proliferation, 2D migration, and invasion into a collagen matrix [14, 18]. Research findings have elucidated PDPN is upregulated by the PI3K-AKT-AP-1 signaling pathway and downregulated by enhancer of zeste homolog 2 (EZH2) and oncogenic mutations IDH1 genes, along with changes in chromatin modifications and DNA methylation, and is closely related to the poor prognosis of gliomas [19]. Moreover, PDPN has been considered as a novel biomarker, chemotherapeutic target and a target for CAR T-cell therapy that may be potential adoptive immunotherapy to treat GBM [20]. Pearson correlation also validated that PDPN was correlated with marker genes of macrophage in gliomas, such as, CD68, etc. [21]. However, the further mechanisms of PDPN in the regulation of the IME and tumor progression in glioma remain largely unclear.

**Fig. 1:**
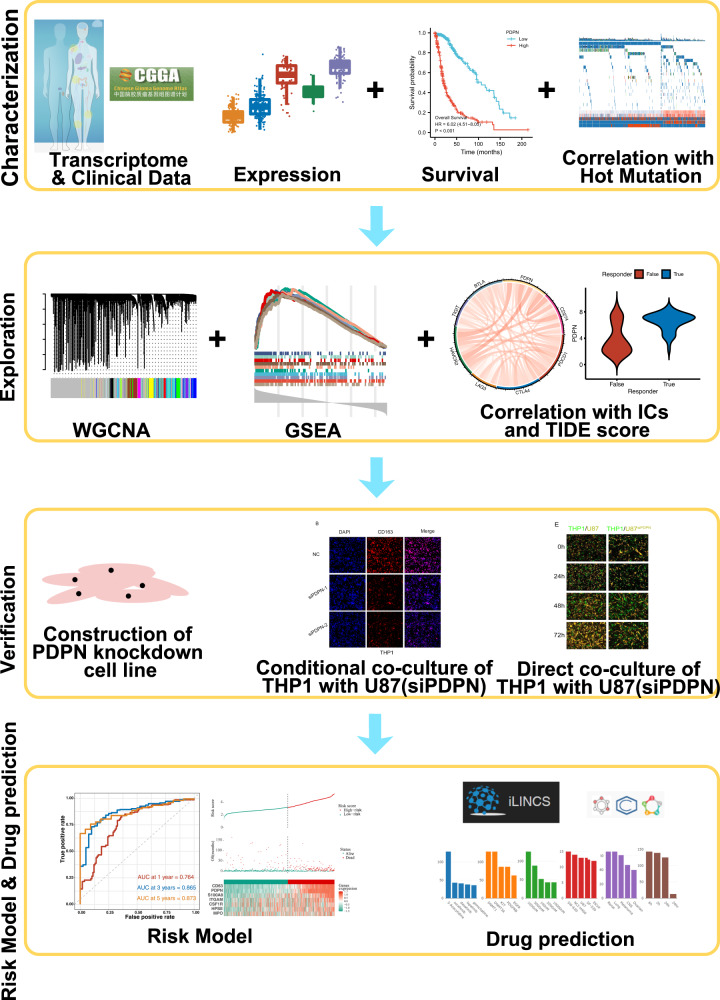
Workflow of the study.

In this study, to investigate PDPN potential mechanisms promoting malignancy of glioma, WGCNA was employed to help discover gene functions and identify disease/phenotype-associated genes in glioma based on TCGA database. Cox and LASSO regression models were also established, which could help us better predict glioma prognosis (Fig. 1).

## Methods and materials

### Data collection

The CGGA (http://www.cgga.org.cn), TCGA (https://xenabrowser.net) and GTEx (https://xenabrowser.net) RNA-Seq datasets were downloaded online. The TCGA mutation dataset was downloaded and analyzed with the help of R package “TCGAbiolinks” [22]. Information on age, gender, WHO grade, primary therapy outcome, patient prognoses, IDH status, 1p/19q status was also collected. Tumor tissues were also collected from glioma patients who underwent surgery at the Tianjin Medical University General Hospital. Written informed consent was obtained from all donors or their relatives. This study was performed in accordance with the principles of the Helsinki Declaration and was approved by the ethical committee of Tianjin Medical University General Hospital.

The neutrophils marker genes and granulation products, M2 macrophages markers, immune checkpoints, M2 macrophage and N2 neutrophils related cytokines were collected from published articles or reviews [23–26].

### Statistical analysis

R language (v. 4.0.2) for Windows was used for statistical analyses and generating figures. GBM and LGG samples from the CGGA and TCGA datasets were analyzed, respectively. Genes with significantly different expression between groups were estimated by a two-tailed Student’s *t*-test or ANOVA (*p value <0.05, **p value <0.01, ***p value <0.001, ****p value <0.0001). We then calculated the prognostic value of PDPN using the “survival” package of R [27]. Univariate and multivariate Cox regression analyses were performed by using the R package “survival” to investigate whether the risk score was an independent OS predictor for glioma patients. Immune cells and genes (neutrophils and macrophages markers, immune checkpoints) correlated with PDPN expression were explored by Pearson’s correlation coefficient (*r*) using R. An absolute *r*-value of greater than 0.4 was considered to indicate a forcefully significant correlation with PDPN. All experimental data were examined at least three times. All the quantitative data are presented as the mean ± SD. The unpaired *t*-test was used to compare the means of two groups.

### Bioinformatic analysis

Weighted Gene Co-expression Network Analysis (WGCNA) was used for finding clusters (modules) of genes highly correlated with PDPN, and for relating modules to external sample traits (IDH-status, ESTIMATE-Immune Scores), and for summarizing intramodular hub genes (MM > 0.9 and GS > 0.7) [28]. Gene Set Enrichment Analysis (GSEA) was performed to identify the biological functions associated with PDPN by the R package “clusterProfiler” [29]. We used Gene Set Variation Analysis (GSVA) to explore the relationship between PDPN and predefined transcriptional profile of neutrophils and macrophages [30]. The responding status of immunotherapy was predicted by TIDE [31]. The drug prediction was performed with iLINCS [32]. The LASSO regression was applied to construct the prognostic with PDPN and the marker genes of neutrophils and macrophages by the R package “glmnet” based on lambda.min [33].The optimal tuning parameter (lambda) was determined through tenfold cross-validations. To calculate the risk score, the expression of each gene in the signature was multiplied by its regression coefficient, and then these values were summed. Survival analysis was applied to assess the predictive value of the signature. Time-dependent receiver operating characteristic (ROC) curve was performed to calculate the area under the curve (AUC) for 1-, 3-, or 5-year overall survival (OS) by using the R package “survivalROC” [34].

### Tissue immunohistochemistry (IHC) and immunofluorescence (IF)

Immunohistochemistry and immunofluorescence were performed using formalin-fixed, paraffin-embedded tissues. Four-μm-thick sections were cut and dewaxed in xylene, rinsed in graded ethanol, and rehydrated in distilled water. After antigen retrieval with EDTA buffer (1 mM Tris/EDTA, pH 9.0), endogenous peroxidase activity was blocked with 3% H2O2. Then slides were incubated with primary antibody (PDPN, 1:100, Abcam, ab236529, USA; CD163, Abcam, ab156769, USA; CD18, Affinity, BF0227, China). For IHC, markers were detected with a Goat Anti-rabbit IgG Two-step Detection Kit (PV-9000, ZSGB-Bio, China). Next, the slides were counterstained with Mayer Hematoxylin Solution (G1080, Solarbio, China) for nuclear staining. For IF, Alexa-Fluor 488 labeled donkey anti-rabbit IgG (Invitrogen, USA, 1:1000) and Alexa-Fluor 594 labeled donkey anti-mouse IgG (Invitrogen, USA, 1:1000) were applied to the double-colored fluorescent staining. Nucleus was labeled by DAPI staining solution (Solarbio, China).

### Cell lines and cell culture

Human glioma cell lines U87MG and U118MG, human leukemia monocytic cell line THP1 were purchased from ATCC (USA). Glioma cell lines were cultured in DMEM (Gibco, USA) and THP1 was in 1640 (Gibco, USA) supplement with 10% FBS, and incubated in 5% CO_2_ at 37 °C. To establish the PDPN-knockdown cell line, siRNA for PDPN was purchased from GenePhama, China: siPDPN-1 (sense: 5’-GUGGCAACAAGUGUCAACATT-3’; antisense: 5’-UGUUGACACUUGUUGCCACTT-3’), siPDPN-2 (sense: 5’-GACCCUGGUUGGAAUCAUATT-3’; antisense: 5’-UAUGAUUCCAACCAGGGUCTT-3’). For conditional co-culture, the first day, add 100 ng/ml PMA into 24-well dished planted with THP1 (1 × 10^5^/well), meanwhile change the medium of U87MG and U87MG-siPDPN to 1640 at 70–80% confluence, after 24 h, collect and filter the 1640 from glioma cell dish to treat THP1 for 24 h. For direct co-culture, firstly, lentiviruses containing red fluorescent gene (RF) and green fluorescent gene (GF) were obtained from GENECHEM and the lentiviruses transduction were performed in U87(-siPDPN) (RF) and THP1 cells (GF). Then, treat the THP1 (GF) (5 × 10^5^) with PMA as above, and add U87(RF) or U87-siPDPN(RF) (5 × 10^5^) for co-culture.

### Real-time polymerase chain reaction (RT-PCR) and western blotting (WB)

The total RNA or protein isolation and subsequent RT-PCR or WB were conducted as previously described [35]. The expression of genes was detected through GoTaq qPCR Master Mix (A6001, Promega, USA). The primers sequences (Genewiz, China) were as follow: PDPN: F 5’-GTGTAACAGGCATTCGCATCG-3’, R 5’-TGTGGCGCTTGGACTTTGT-3’; GAPDH: F 5’-GGTGGTCTCCTCTGACTTCAACA-3’, R 5’-GTTGCTGTAGCCAAATTCGTTGT-3’; CSF1: F, CGCCCACTCCGCAGC, R CCAGCCATGTCGTGGGAG; CSF2: F TTCCTGCTCAAGTGCTTAGAG, R AGCTTGTAGGTGGCACAC; CSF3: F CTGAACCTGAGTAGAGACACTG, R GCCCTTGAGCTTGGTGAG; CCL-2: F TCTGTGCCTGCTGCTCATAG, R GGGCATTGATTGCATCTGGC; IL-10: F CGCATGTGAACTCCCTGG, R TAGATGCCTTTCTCTTGGAGC; TGF-β: F GTGGTATACTGAGACACCTTGG, R CCTTAGTTTGGACAGGATCTGG; CXCL2: F AACCGAAGTCATAGCCACAC, R CTTCTGGTCAGTTGGATTTGC; CXCL-5: F TCTGCAAGTGTTCGCCATAG, R CAGTTTTCCTTGTTTCCACCG. The primary antibodies for WB: PDPN (Abcam, ab236529), IL-10 (Abclonal, A2171), CSF1 (Abclonal, A1627), TGFB1 (Abclonal, A2124), CSF2 (Abclonal, A6127), CSF3 (Abclonal, A6178), CXCL2 (Abclonal, A12639). Data were analyzed using the relative standard curve method and normalized to GAPDH or TUBULIN.

### Flow cytometry

First, THP1 cells were treated as mentioned above (cell lines and cell culture), then digested with accutase, collected, washed for three times with PBS. Then incubated with antibody mixture for 30 min (Brilliant Violet 421™ anti-CD163, BioLegend, 333611; FITC anti-CD86, BioLegend, 374203), washed with PBS again. A Bioscience FACScan Flow Cytometry System (BD Biosciences, Franklin Lake, NJ, USA) was used to detect markers expression.

## Results

### PDPN is highly expressed in IDH-WT GBM’s at mRNA and protein levels

**Fig. 2:**
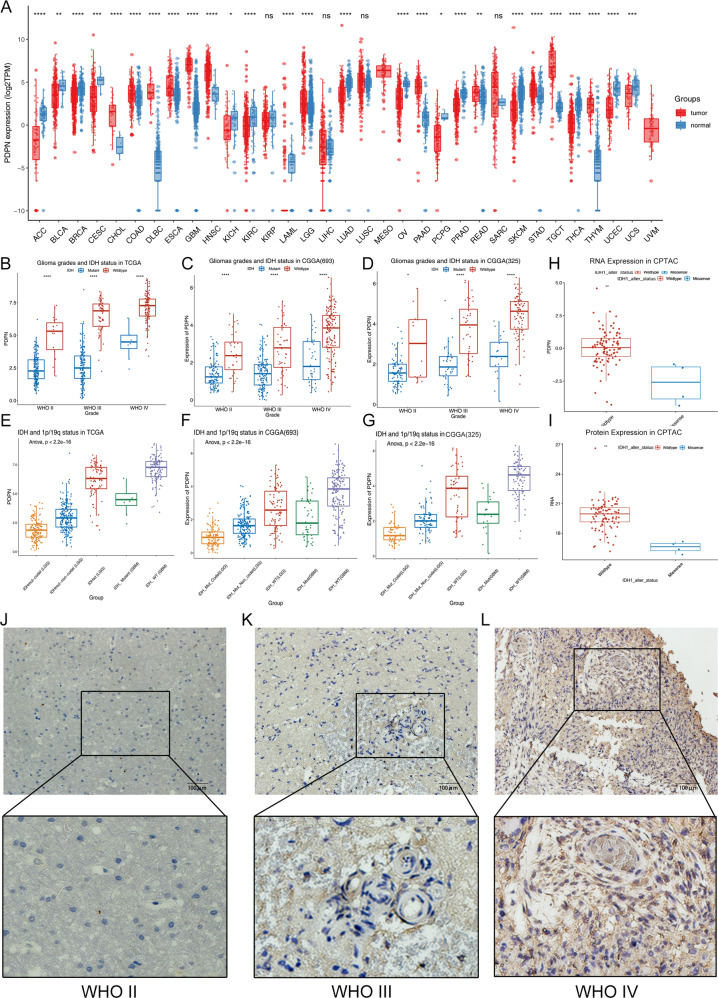
PDPN is highly expressed in IDH-WT GBMs at mRNA and protein levels. **A** The expression profile of PDPN in 31 kinds of cancers and their paired normal tissues from TCGA database. **B**–**D** The relationship between PDPN mRNA expression and WHO glioma grades and IDH mutation status of glioma samples in the TCGA and CGGA databases. **E**–**G** The expression of PDPN in IDH-Mut-co-del LGG, IDH-Mut-non-co-del LGG, IDH-WT LGG, IDH-Mut GBM, and IDH-WT GBM. **H**, **I** PDPN showed lower expression both at mRNA and protein levels. **J**–**L** Higher PDPN level is observed with increasing tumor grade in glioma clinical samples (ns *p* > 0.05, *p < 0.05, **p < 0.01, ***p < 0.001, ****p < 0.0001).

First, we analyzed the expression profile of PDPN in pan-cancer. The PDPN expression values in 33 kinds of cancers were extracted from TCGA database and compared with the PDPN expression values in the tissues of non-lesion sites obtained from GTEx database of each cancer type (Fig. 2A). Among 33 cancers, MESO and UVM did not match the corresponding normal tissue. In the remaining 31 cancers, there was no statistically significant difference in the expression changes of PDPN in 4 cancers (KIRP, SARC, LUSC, LIHC). PDPN expression was significantly downregulated in 14 cancers (ACC, BLCA, BRCA, CESC, KICH, KIRC, LUAD, OV, PCPG, PRAD, SKCM, THCA, UCEC, UCS), and significantly upregulated in 13 cancers (CHOL, COAD, DLBC, ESCA, GBM, HNSC, LAML, LGG, PAAD, READ, STAD, TGCT, THYM). PDPN was significantly upregulated in both GBM and LGG (GBM vs GTEX, Log2 Foldchange = 5.01, *p* = 0; LGG vs GTEx, Log2 Foldchange = 2.07, *p* = 3.59 × 10^−30^) (Fig. 2A).

Furthermore, we performed a stratified analysis based on the WHO classification and IDH mutation status of glioma samples in the TCGA and CGGA databases. We found that PDPN mRNA expression increased with the tumor grade, and the expression in IDH-wildtype (IDH-WT) gliomas of each grade was higher than that in IDH-mutant (IDH-Mut) gliomas. Importantly, the PDPN had the highest expression level in IDH-WT GBM (Fig. 2B–D). Adding 1p/19q status analysis, we found that the status of 1p/19q was also correlated with the PDPN mRNA expression level. Compared with LGG with IDH mutation and 1p/19q non-deletion (IDH-Mut-non-co-del, LGG), the PDPN mRNA expression was lower in LGG with IDH mutation and 1p/19q co-deletion (IDH-Mut-co-del, LGG) (Fig. 2E–G). These results suggested that PDPN may be preferentially expressed in astrocytoma. Moreover, we also found that the PDPN mRNA expression level in IDH-WT LGGs was only inferior to IDH-WT GBMs and higher than any other types, including IDH-Mut GBMs (Fig. 2E–G). We further conducted studies using the CPTAC dataset and confirmed the low expression of PDPN in IDH1 mutant samples, at mRNA as well as protein level (Fig. 2H, I). These results suggest that PDPN is closed related to IDH status and is preferentially expressed in IDH-WT GBMs.

To further confirm the expression level of PDPN in gliomas, we took advantage of clinical human glioma samples to determine the relationship between the expression of PDPN in glioma tissues and glioma grades. The expression of PDPN was abundant in WHO grade IV glioma tissues and lower expression levels in WHO grade III glioma tissues, especially in the vascular zone. However, it was hardly detected in WHO grade II glioma tissues (Fig. 2J–L).

### Upregulated PDPN expression is correlated with the poor prognosis in glioma patients

**Fig. 3:**
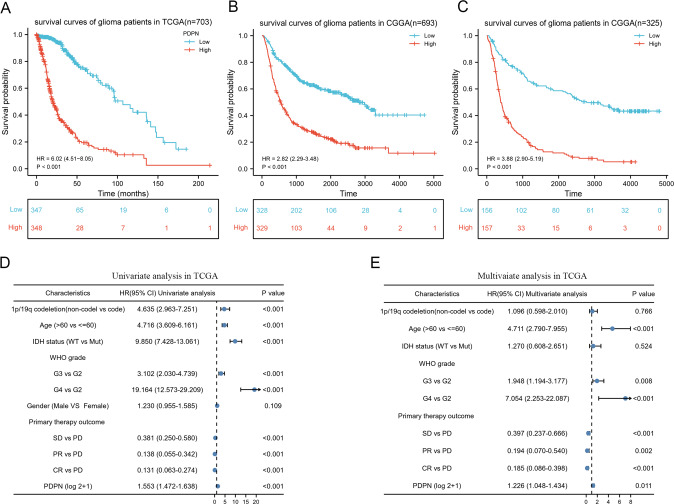
Upregulated PDPN expression is correlated with the poor prognosis in glioma patients. **A**–**C** The TCGA and CGGA datasets were used for survival analysis of the two groups of glioma patients with higher and lower PDPN expression in glioma patients. **D**, **E** Univariate and multivariate Cox regression analyses to evaluate the correlation of PDPN expression with prognosis of glioma patients in the TCGA databases.

To determine the prognostic value of PDPN gene expression in glioma patients, Kaplan–Meier survival curves were performed using data from the TCGA and CGGA clinical information, RNA-seq datasets. The result showed that the overall survival (OS) time of glioma patients with higher PDPN expression were shorter than glioma patients with lower PDPN expression in the TCGA RNA-seq database (*p* < 0.001) (Fig. 3A). Moreover, in the CGGA RNA-seq database (*n* = 693 and *n* = 325), glioma patients with higher PDPN expression were also connected with a worse prognosis than those with lower PDPN expression (*p* < 0.001) (Fig. 3B, C).

To further confirm our conjecture, univariate cox analysis was employed (samples included LGG and GBM in TCGA database). The analysis unearthed that PDPN was a high-risk factor (HR = 1.553; 95% CI = 1.472–1.638; *p* < 0.001). Besides, WHO grade, age, 1p/19q co-deletion, IDH status, primary therapy were all independent prognostic factors. Then multivariate analysis was performed, and it was found that among these factors, PDPN (HR = 1.226; 95% CI = 1.048–1.434; *p* < 0.05) remained independently related to overall survival, suggesting that PDPN could be an independent prognostic factor for glioma patients (Fig. 3D, E).

### High PDPN expression is closely associated with hallmark gene variations in glioma

**Fig. 4:**
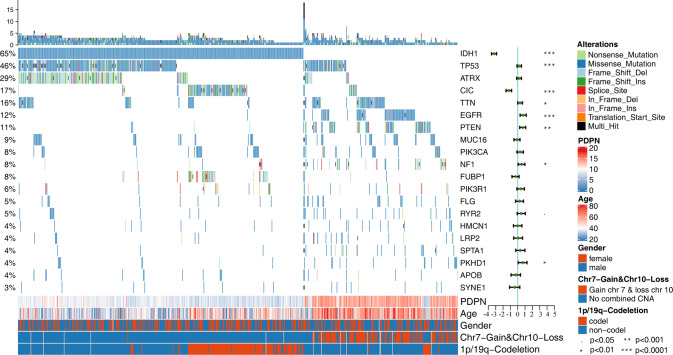
High PDPN expression is closely associated with hallmark gene variations in glioma. Multivariable regression analysis of the top 20 mutation frequencies genes, age, chromosome 7 acquisition, chromosome 10 deletion and 1p/19q combined deletion and PDPN expression level (*p* < 0.05, *p < 0.01, **p < 0.001, ***p < 0.0001).

Multiple linear regression analysis was performed on the mutation status of the genes with the top 20 mutation frequencies in glioma and the expression level of PDPN. IDH1, TP53, CIC, PTEN, EGFR showed significant indigenous correlation with PDPN. Among them, the mutations of IDH1 and CIC were significantly negatively correlated with PDPN, while PTEN, TP53, and EGFR were significantly positively correlated. In addition, the trait data of the samples, including age, chromosome 7 acquisition, chromosome 10 deletion and 1p/19q combined deletion. Older age and Chr 7 Gain and Chr 10 Loss associated with higher PDPN expression. While 1p/19q existed opposite to PDPN overexpression (Fig. 4). Partial results such as the IDH1 and EGFR, are consistent with our first part and previous research findings [14, 19]. The CIC and 1p/19q associated with PDPN expression reasonably for the PDPN genomic location (1p36.21). The Chr 7 Gain may affect PDPN expression by upregulate the EGFR-PI3K pathway. However, the mechanism behind the relationship between TP53 mutation and PDPN remains to be known.

### PDPN is closely related to immune microenvironment

**Fig. 5:**
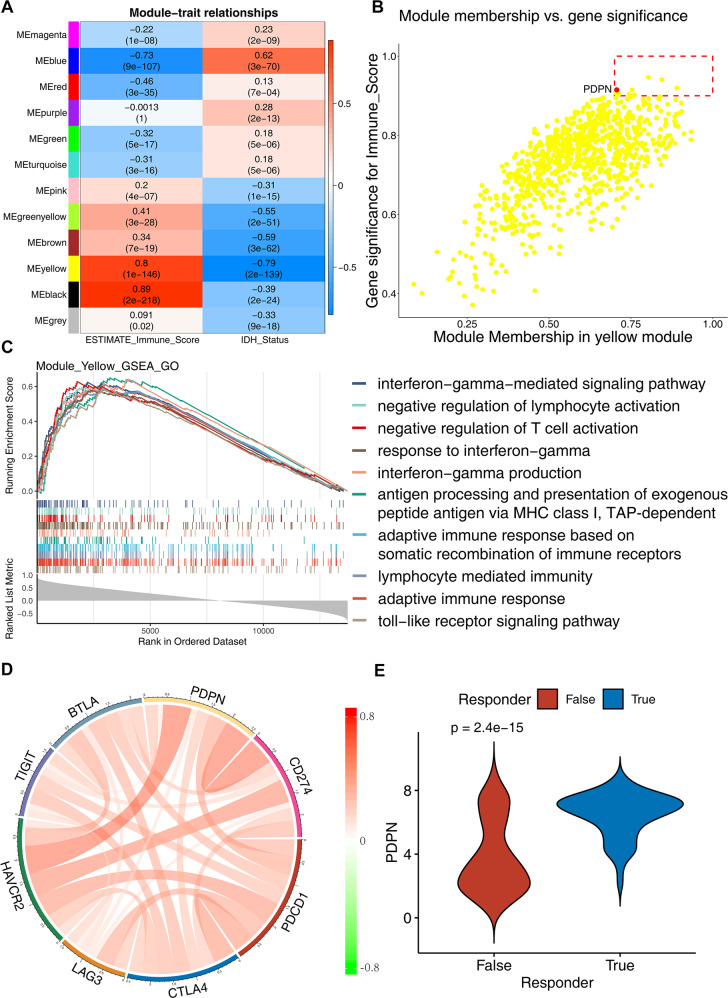
PDPN is closely related to immune microenvironment. **A** The correlation between IDH mutation status and ESTIMATE-immune score of this module. **B** The correlation of genes in Yellow Module and Immune Score. **C** GSEA functional enrichment analysis of Yellow Module genes. **D** Correlation of PDPN and immune checkpoint molecules in glioma of TGGA dataset. **E** PDPN expression was related to responding status to immnotherapy predicted by TIDE.

We performed WGCNA analysis of GBMLGG in TCGA database, to analyze the function of PDPN-related modules through this optimized gene clustering method. The first was the soft threshold screening, and 15 got defined as the best soft threshold. Based on this, a total of 14140 protein code genes were divided into 12 modules. We found that PDPN was classified into the yellow module. We analyzed the correlation between IDH mutation status and ESTIMATE-immune score of this module and found that the yellow module where PDPN located was significantly negatively correlated with IDH mutation status (Cor = −0.79, *p* < 0.01), and positively correlated with immune score (Cor = 0.8, *p* < 0.01) (Fig. 5A). Further analysis of the correlation between genes in the yellow module (defined as Module Membership) showed that genes highly correlated with the module were also highly correlated with ‘immune scores’ trait (defined as Gene Significance), and PDPN was in the core area of the module (MM > 0.9 and GS > 0.7 (Fig. 5B), suggesting that PDPN played a key role in junction between the module and immune trait. We continued to perform gene set enrichment analyses (GSEA), where we tested for the yellow module among gene ontology (GO) – biological processes (BP) terms. It was found that this module was highly enriched with adaptive immunity, negative regulation of T cell activation and negative regulation of lymphocyte activation (Fig. 5C). Then we also found that PDPN was significantly positively correlated with the expression of 7 immune checkpoints, especially CD274 and HAVCR2 (Fig. 5D). We submitted the expression sequences of all glioma patients from TCGA to the TIDE website for analysis and found that PDPN expression was higher in those samples predicted by TIDE to respond to immunotherapy than in samples that did not respond (Fig. 5E). Therefore, we speculated that PDPN plays an important role in the construction of glioma immune microenvironment.

### PDPN closely associates with neutrophils degranulation and M2 macrophages polarization

**Fig. 6:**
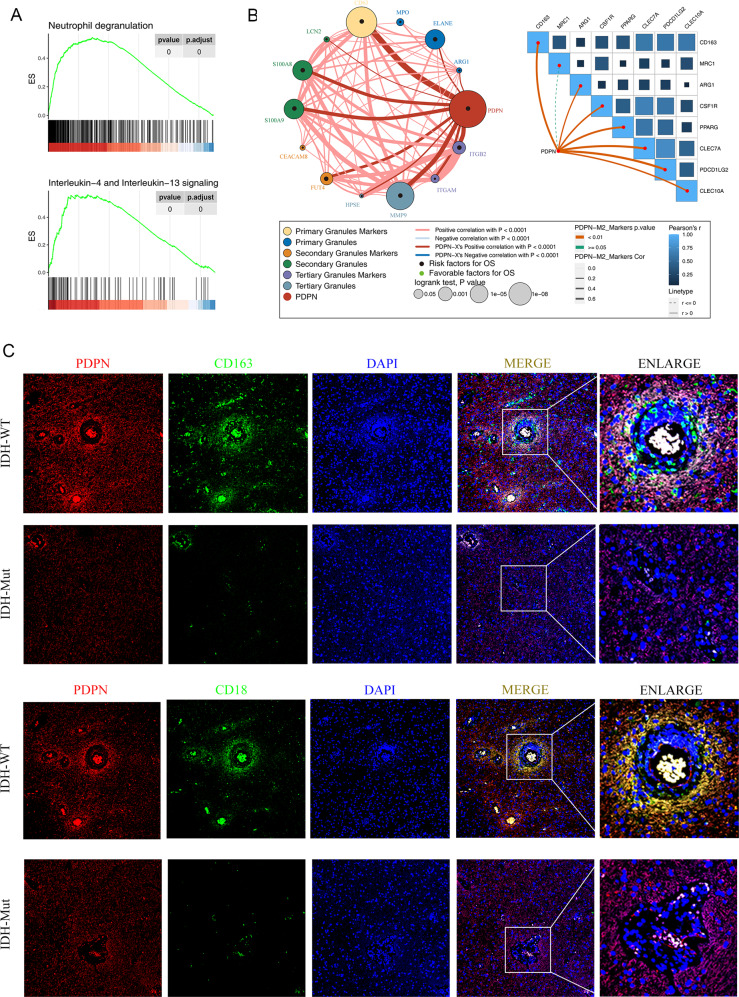
PDPN closely relates to neutrophils and macrophages in glioma immune microenvironment. **A** The yellow module was highly enriched in neutrophil degranulation and Interleukin-4 and Interleukin-13 signaling. **B** PDPN closely related with neutrophil markers, degranulation products, and macrophage markers. **C** Double-colored fluorescent staining showed PDPN spatially co-expressed with CD18 and CD163.

We performed GSEA analysis based on Reactome pathway databases, showing that the yellow module was closely correlated with neutrophil degranulation and interleukin-4 and interleukin-13 signaling (Fig. 6A). Glioma cell have been illuminated to gang up on M2 macrophages which could be induced by IL4 and IL13 to invade the immune surveillance, especially in GBMs [36]. Besides, growing number of studies have reported the fact that tumor-associated neutrophils could also promote the tumor malignancy [37]. Thus, we got the markers during the process of neutrophil degranulation and M2 macrophages. Then correlation analysis was carried out. Results showed that there was a significant correlation between neutrophil markers (CD63, FUT4, ITGAM, ITGB2), and degranulation products (ELANE, S100A8, S100A9, MMP9). In addition, PDPN expression positively correlated with most macrophage markers (CD163, ARG1, CSF1R, PPARG, CLEC7A, CEACAM8, PDCD1LG2, CLEC10A), all correlation coefficient ≥0.04 and *p* value <0.01 (Fig. 6B). To further confirm these initial findings, tumor section from a GBM patient was co-immuno-stained with tertiary granules marker ITGB2 (CD18) and M2 macrophage marker CD163 and observed via confocal microscopy (Fig. 6C). We found that PDPN was highly stained, especially at perivascular area, which was consistent with the IHC. Furthermore, the perivascular areas displayed a higher spatial co-expression density of PDPN, CD18, CD163, which may suggest that PDPN mainly expressed by GBM cell may promote CD18^+^ and CD163^+^ immune cell infiltration.

### Vitro verification shows PDPN was closely related to the infiltrations of TAMs and TANs

**Fig. 7:**
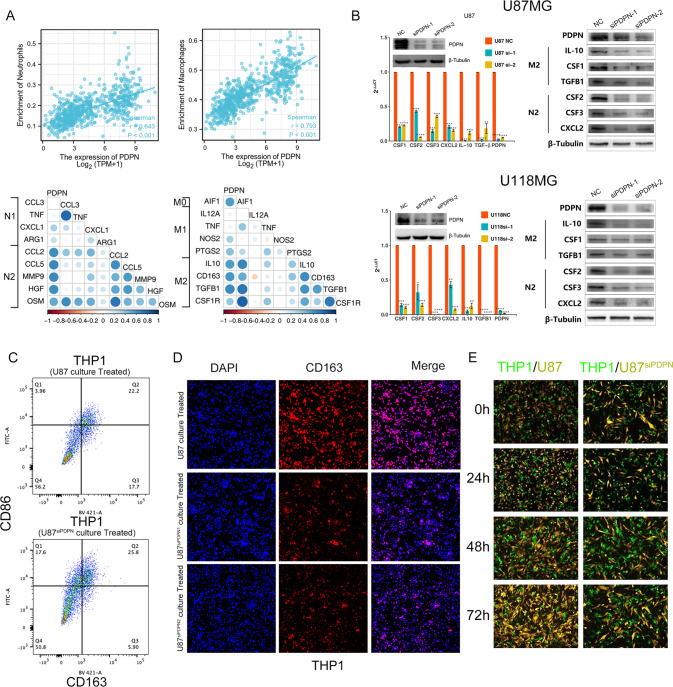
PDPN knockdown reduced the ability of GBM cell to induce TAM and TAN infiltration and polarization. **A** Neutrophils and macrophages were significantly positively correlated with PDPN level. **B** Decreased expression of M2 TAM and N2 TAN related cytokines was consistent with knockdown of PDPN. **C**, **D** Flow cytometry and IF verified the knockdown of PDPN damaged the ability of U87MG to induce CD163 expression of THP1. **E** Co-culture of U87 (siPDPN) with THP1.

First, we performed further analysis aiming to seek for the correlation between PDPN expression levels with neutrophils and macrophages. As expected, it showed that neutrophils and macrophages were significantly positively correlated with PDPN level (Cor = 0.643, *p* < 0.001; Cor = 0.793, *p* < 0.001) (Fig. 7A). Furthermore, PDPN showed higher correlation with markers of N2 and M2 phenotypes. To verify our analyses, two GBM cell lines (U87MG and U118MG) from CCLE with high PDPN expression were selected and used to establish PDPN-knocked down cell models (Fig. 7B). We detected the changes of mRNA expression of both M2 type TAM related cytokines (CSF1, IL-10, TGF-β) and N2 type TAN related cytokines (CXCL2, CSF2, CSF3) secreted by GBM cells through qPCR and WB. Interestingly, all these cytokines were markedly decreased in PDPN-knockdown GBM cell lines (Fig. 7B). Subsequently, we treated THP1 cells with 100 ng/ml of PMA firstly, and then cultured with U87MG and U87MG-siPDPN cell culture medium for 24 h, and finally detected the expression of CD163 by immunofluorescence. Notably, the expression of CD163 in U87MG-siPDPN medium cultured THP1 cell was significantly lower than that in U87MG group (Fig. 7C, D). When U87 cells were co-cultured with induced THP1 cells for 72 h, we found that the siPDPN group had slow proliferation, indicating that PDPN knockdown affected the mutual benefit between tumor cells and macrophages (Fig. 7E). Collectively, we found that PDPN knockdown reduced the ability to induce TAM and TAN infiltration and polarization toward M2 or N2 type in vitro, which has not been reported by now.

### PDPN-related markers construction of the prognostic model

**Fig. 8:**
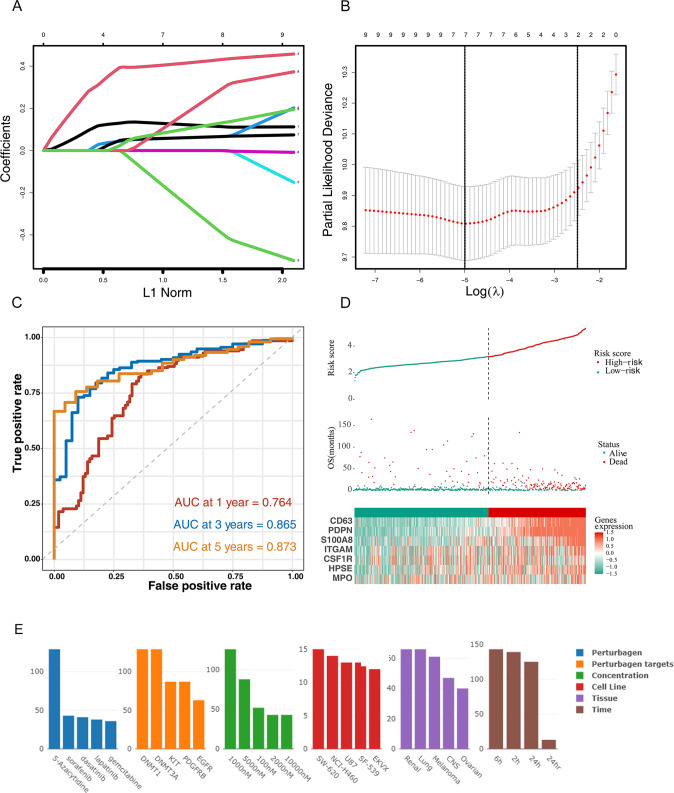
Construction of the prognostic model. **A**, **B** Lasso-Cox analysis of 9 PDPN-related markers. **C**, **D** Time-dependent ROC curve and the risk score distribution stratified by CD63, PDPN, LCN2, PDCD1LG2, ITGAM, CSF1R, MPO, HPSE expression levels in the TCGA dataset. **E** Drugs prediction to reverse the expression pattern in high-rigk group.

Univariate and multivariate Cox regression were performed in order among the total 20 neutrophil degranulation and macrophage markers (PDPN, CD63, CSF1R, S100A8, S100A9, CEACAM8, MPO, ITGAM, HPSE, FUT4, ARG1, PDCD1LG2, ELANE, CLEC7A, ITGB2, MMP9, CD163, CLEC10A, PPARG, LCN2) to identify genes of significant correlation with OS (Supplementary Fig. 1) and 8 genes with *p* < 0.05 were selected into LASSO regression for further shrinkage (CD63, PDPN, PDCD1LG2, LCN2, ITGAM, CSF1R, HPSE, MPO). Upon the partial likelihood deviance reaching minimum in the LASSO regression, all genes were identified and selected to construct the prognostic model (Fig. 8A, B). The formula for calculating the risk score was: PDPN * 0.26440192 + CD63 * 0.43070903 + CSF1R * −0.64435283 + PDCD1LG2 * 0.11738595 + MPO * 0.06863899 + ITGAM * 0.35244738 + HPSE * 0.19819484 + LCN2 * 0.06200738.

The median of the risk score of the prognostic model was used for cut-off value and patients were classified into low- and high-risk groups accordingly. Furthermore, we assessed the prognostic efficiency of the 8 genes model by operating a ROC curve and the AUCs for 1-, 3- and 5-year OS were 0.887, 0.916, 0.870, respectively (Fig. 8C, D). We then submitted the top 150 genes that were highly expressed in the high-risk group to the ILINCS website, hoping to find perturbagens that could reverse the expression of 150 genes (Fig. 8E). The 5-Azacytidine targeting DNMT1 took first place to show potential to reverse the expression pattern. The following drugs include some RTKs inhibitors like sorafenib, dasatinib and lapatinib, which are promising candidates for patient benefit by reversing the effects of PDPN on the immune microenvironment.

## Discussion

PDPN has been associated with tumor cell migration and proliferation in vitro, but the tumor progression in a preclinical mouse model occurs independently of PDPN [18, 38]. Notably, the SCID-beige animals used in the zoopery carry profound defects in natural killer cells and the adaptive immune system [39]. We demonstrate that PDPN is involved in the formation of glioma progression IME: GSEA results in our study suggested PDPN correlated gene set highly enriched the adaptive immunity, negative regulation of T cell activation and negative regulation of lymphocyte activation (Fig. 5E), and neutrophil degranulation and Interleukin-4 and Interleukin-13 signaling (Fig. 6A, B). Correlation analysis showed PDPN was closely related with M2 macrophage markers, like CD163, ARG1, etc. and neutrophil markers and degranulation products, like CD18, MMP9. The IF verified it that CD163 and CD18 highly expressed cell co-localized with PDPN highly expressed cells. But there comes a meaningful question: whether the association of PDPN with these immune cells is due to a direct effect, or the associated microthrombotic event? Or both? On one hand, given that platelet-derived-growth factor (PDGF) has been reported to promote macrophages survival and polarization, neutrophils activation and secretion [40], we proposed PDPN can aggregate and activate platelets in glioma, allowing cytokines secretion to form the IME. Platelets contain and secrete many mediators involved in hemostasis and inflammation [41]. Activated platelets release IL-1, which plays a major role in the inflammatory cytokine cascade and TGFβ, which is well known as a key mediator of GBM-induced immunosuppression [42–45]. Lately research that revealed deletion of PDPN in glioma cells resulted in a significant reduction of intratumoral platelet aggregates in vivo preliminarily confirmed the hypothesis [46].On the other hand, herein, we found that PDPN knockdown can directly reduce the ability of GBM cell to induce TAM and TAN infiltration and polarization toward M2 or N2 type in vitro, which has never been reported before. Perturbagens like 5-Azacytidine and RTKs inhibitors showed the ability to reverse the high-risk gene expression pattern of PDPN risk model, improve the immune microenvironment, especially reduce the gangrene with macrophages. The underlying regulation mechanism remains to be explored and we suggest here that the value of targeting PDPN can be fully reconsidered in terms of improving the GBM IME.

In conclusion, we found that PDPN may participate to construct the IME in glioblastoma. Further exploration will be carried out to reveal the underlying mechanism. We also found that a gene community which has highly expression in IDH wildtype gliomas and is significantly associated with immune scores, and we sincerely recommend further analysis and validation of these genes to provide guidance and help for targeted therapy and immunotherapy of glioma.

## Supplementary information

Supplementary Figure 1

## Data Availability

Data available in a publicly accessible repository, including CGGA (http://www.cgga.org.cn), TCGA (https://xenabrowser.net) and GTEx (https://xenabrowser.net).
