# Supplementary material for: PDPN contributes to constructing immunosuppressive microenvironment in IDH wildtype glioma
Source: Cancer Gene Ther. 2022 Nov 25;30(2):345–57. doi: 10.1038/s41417-022-00550-6 (PMC9935394; doi:10.1038/s41417-022-00550-6)
Supplement: Supplementary file 1 — Supplementary Figure 1 [file 41417_2022_550_MOESM1_ESM.pdf]

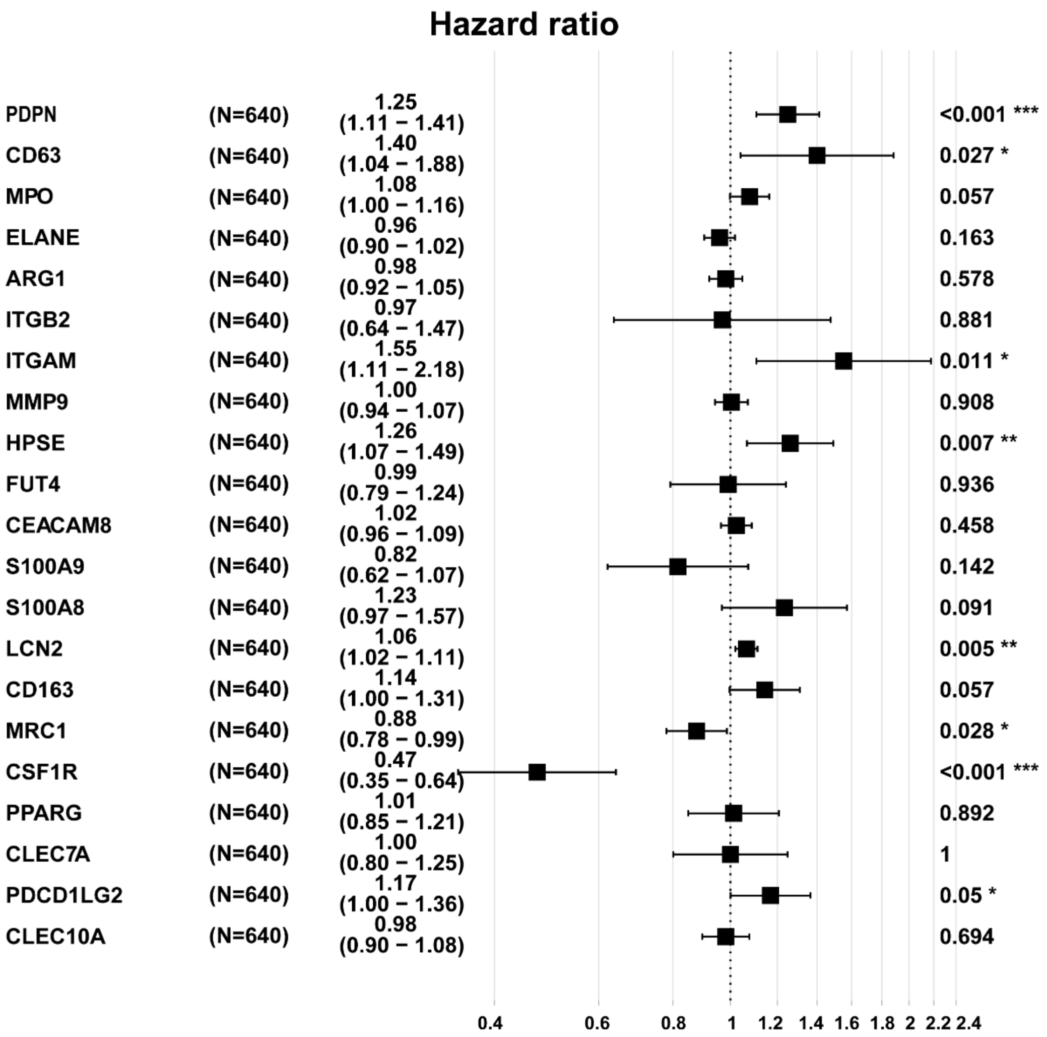

Supplementary Figure 1. Multivariate Cox regression of PDPN and the 20 neutrophil degranulation and macrophage markers genes
